# Internet safety education for youth: stakeholder perspectives

**DOI:** 10.1186/1471-2458-13-543

**Published:** 2013-06-05

**Authors:** Megan A Moreno, Katie G Egan, Kaitlyn Bare, Henry N Young, Elizabeth D Cox

**Affiliations:** 1 School of Medicine and Public Health, University of Wisconsin, Madison, WI, USA; 2 School of Nursing, University of Wisconsin, Madison, WI, USA; 3 School of Pharmacy, University of Wisconsin, Madison, WI, USA; 4 Seattle Childrens Research Institute, University of Washington, M/S CW8-6 PO Box 5371, Seattle, WA 98145-5005, USA

**Keywords:** Internet safety, Online safety, Parent education, Patient education, Survey research, Pediatrics

## Abstract

**Background:**

Internet use is nearly ubiquitous among US youth; risks to internet use include cyberbullying, privacy violations and unwanted solicitation. Internet safety education may prevent these negative consequences; however, it is unclear at what age this education should begin and what group is responsible for teaching this topic.

**Methods:**

Surveys were distributed to key stakeholders in youth safety education including public school teachers, clinicians, parents and adolescents. Surveys assessed age at which internet safety education should begin, as well as experiences teaching and learning internet safety. Surveys of adults assessed willingness to teach internet safety. Finally, participants were asked to identify a group whose primary responsibility it should be to teach internet safety.

**Results:**

A total of 356 participants completed the survey (93.4% response rate), including 77 teachers, 111 clinicians, 72 parents and 96 adolescents. Stakeholders felt the optimal mean age to begin teaching internet safety was 7.2 years (SD = 2.5), range 2-15. Internet safety was regularly taught by some teachers (20.8%), few clinicians (2.6%) and many parents (40.3%). The majority of teachers, clinicians and parents were willing to teach internet safety, but all groups surveyed identified parents as having primary responsibility for teaching this topic.

**Conclusions:**

Findings suggest agreement among key stakeholders for teaching internet safety at a young age, and for identifying parents as primary teachers of this topic. Clinicians have a unique opportunity to support parents by providing resources, guidance and support.

## Background

While the internet has provided adolescents with numerous benefits, including increased social support, academic enrichment and worldwide cross-cultural interactions, there are concomitant risks to internet use [1-8]. The American Academy of Pediatrics’ (AAP) recent report on children’s social media use describes specific risks such as privacy violations and cyberbullying [9]. A previous study found that one-third of adolescents had given their internet password to friends and one-fourth were unaware that content uploaded online cannot be permanently deleted [1]. Cyberbullying, or internet harassment, impacts up to a third of youth and has been linked to a variety of health concerns, some as serious as suicidal ideation [10-15]. In addition, adolescents frequently display personal and identifiable information about themselves on the internet. These details may include their home location, revealing photographs, or descriptions of sexual behavior and substance use [16-18].

Internet safety is highly salient for today’s youth as they spend up to 10 hours a day using various forms of media [8,19,20]. The ever-increasing popularity of social media, including websites such as Facebook and Twitter, have contributed to youth’s time investment in the internet [7]. The vast majority of adolescents have internet access and most report daily use [21,22]. Several organizations, including the AAP, have offered expert advice regarding internet safety, but an evidence-based approach to educate youth about the dangers of being online does not currently exist [23]. Further, data to guide decisions about the age at which such education should begin, and who would have primary responsibility for teaching this topic are incomplete.

An ideal approach for teaching internet safety would likely involve a person or group who could reach most children in order to provide widespread dissemination of this knowledge. An ideal candidate would also have experience teaching about the internet or related safety issues, and be willing to invest in teaching this topic. Given that most US youth and adolescents attend public school, a first possibility is public school teachers. However, it is unclear at what grades and in which school subjects this material could be integrated into existing curricula. A second possibility is child health providers such as pediatricians or family medicine physicians. The AAP social media report argues that “pediatricians are in a unique position” to provide internet safety education [9]. Several resources exist to guide pediatricians in these discussions, but it is unclear whether pediatricians are comfortable in these discussions. Previous work has suggested that pediatrician’s performance of adolescent health behavior screening and prevention counseling regarding health risk behaviors is quite low [24,25]. A third potential candidate is the parent of the adolescent. While adults’ use of online media such as social networking sites continues to rise, data regarding parents’ comfort or experience with teaching internet safety remains elusive [26]. While all three groups undoubtedly should play a role in online safety education, it remains unclear which group is seen as holding primary responsibility among these stakeholders.

The purpose of this study was to investigate views of key stakeholders on internet safety education, including school teachers, clinicians who see children and adolescents, parents of adolescents, and adolescents themselves. Our goals were to investigate at what age internet safety education should begin, and to identify a primary candidate to teach this topic.

## Methods

This study was conducted between July 1, 2009 and August 15, 2011 and received IRB approval from the University of Wisconsin Human Subjects Committee.

### Setting and subjects

Participants in this study included public school teachers, health care providers who see children and adolescents, parents of adolescents, and adolescents themselves. School teachers were recruited from a summer continuing education conference within a public school district. This district includes 4 elementary schools, one middle and one high school. Inclusion criteria limited participants to teachers who taught kindergarten through 12^th^ grade within that public school district. Clinicians were recruited at a yearly regional continuing medical education conference; inclusion criteria limited participants to physicians (MDs and DOs), nurse practitioners (NPs), physician assistants (PAs), and nurses, all of whose practice included pediatric patients. Parents of adolescents were identified within a large general pediatric practice that includes 8 pediatric providers. Inclusion criteria for parents were that they had a child between the ages of 11 and 18 years. Adolescents (ages 11-18 years) were identified and recruited within this same large general pediatric practice. Most parents and teens were recruited as dyads. We did not exclude parents or teens who elected to participate in the study separately because we did not compare data between parents and teens.

### Data collection and recruitment

In each recruitment setting, potentially eligible participants were approached by a research assistant. After explaining the study and obtaining consent, participants completed a paper survey. Survey respondents were provided a $5 gift card as compensation.

### Survey design

The goals of the survey were to understand at what age internet safety education should begin, explore the experiences of adult participants in teaching online safety or the adolescents learning about this topic, and to identify a group who has primary responsibility for teaching this topic. Thus, we included all potential survey participants in the survey design process. Surveys were designed after a review of the literature and conversations with a panel of physicians, parents and researchers. Questions were pilot-tested first with a panel and then among teachers and adolescents. In the final survey items some words were modified to make the survey clear to all groups of participants. For example, among health care provider groups the question: “For how many years have you been in practice?” was changed for teacher groups to read: “For how many years have you been teaching?” All four surveys are included as Additional files 1, 2, 3 and 4.

### Data sources and variables

Participants provided demographic data including gender and age. Teachers were asked to disclose the grade levels they taught, subjects taught and years of teaching experience. Clinicians were asked to provide their training background (i.e. MD, NP), field of practice (Pediatrics, Family Practice) and years in practice. Parents provided their age, gender and the ages of their children. Adolescents were asked for their age, gender and grade in school.

#### Age to begin teaching internet safety

Teachers, clinicians, parents and adolescents were asked to provide at what age internet safety education should begin. An “other” option was presented for write-in answers.

#### Candidates to teach internet safety

In order to identify potential candidates to teach internet safety, participants were asked about previous experiences teaching or learning about internet safety. Then participants were asked for their own willingness to teach this subject and to identify an ideal primary candidate to teach this topic.

#### Experiences teaching internet safety

To describe experiences in providing internet safety education, teachers were asked how frequently they had ever taught internet safety education. Clinicians were asked how frequently they had ever counseled patients on this topic. Answer options included regularly, sometimes, never and never but plan to do so soon. Parents were asked about how frequently they talked with their child about internet safety: regularly, sometimes, never and never but plan to do so soon (Table 1).

**Table 1:** Demographic data

	**N (%)**
**Teachers**	n = 77
*Teaching site*	
Elementary	44 (57.1%)
Middle	11 (14.3%)
Elementary & Middle	2 (2.6%)
High	20 (26.0%)
*Subject taught*	
Technology or computers	19 (24.7%)
Health	10 (12.9%)
Guidance	2 (2.6%)
Library	4 (5.2%)
Social Studies or History	11 (14.3%)
Language or Communication Other	28 (36.4%) 3 (3.9%)
**Clinicians**	n = 111
*Provider type*	
MD/DO	68 (61.3%)
RN	8 (7.2%)
NP	16 (14.4%)
PA	15 (13.5%)
Other	4 (3.6%)
*Field*	
Pediatrics	68 (61.3%)
Family Practice	31 (27.9%)
OB/Gyn	1 (0.9%)
Other	11 (9.9%)
**Parents**	n = 72
*Relationship*	
Mother	59 (81.9%)
Father	13 (18.3%)
**Adolescents**	n = 96
*Gender*	
Male	36 (37.5%)
Female	60 (62.5%)

#### Adolescents’ experiences learning about internet safety

Adolescents were asked ways in which they had learned about internet safety. A list of answer options was developed through review of the literature and the web and then piloted with several adolescents to ensure completeness. Answer options included learning from friends, siblings, parents, teachers and clinicians as well as learning by self-teaching. A write-in “other” option was also provided. Adolescents were allowed to choose all applicable answers from this list.

#### Willingness to teach internet safety

Teachers were asked whether or not they supported teaching internet safety education in public schools. Health care providers were asked whether or not they supported teaching internet safety education in provider offices (yes or no).

All groups, including teachers, clinicians, parents and adolescents were asked to select a candidate group whom they felt had primary responsibility for teaching internet safety to children and adolescents. Based on a review of current groups engaged in teaching this subject, answer options included churches, community groups, health care providers, law enforcement, parents and teachers. An “other” option was presented for write-in answers.

### Analysis

All statistical data analyses were conducted using STATA version 11.0 (Statacorp, College Station, TX). Descriptive statistics were calculated for survey responses. ANOVA was used to compare mean age to begin teaching between teachers, clinicians, parents and adolescents. Logistic regression was used to assess whether experience teaching internet safety was associated with years of career experience.

## Results

### Participants

A total of 356 participants completed the survey (93.4% response rate), including 77 teachers, 111 clinicians, 72 parents and 96 adolescents. Teachers had an average of 14.8 (SD = 8.4) years of teaching experience. The subjects that teachers taught included: health, social studies, language arts/English, special education, health and technology/computer skills. Clinicians included 68 (61.3%) physicians, 16 (14.4%) nurse practitioners, 15 (13.5%) physician assistants and 8 (7.2%) nurses. Their practice background was mainly pediatrics (61.3%) and family practice (27.9%). Clinicians’ years of experience averaged 14.5 (SD = 10.1). Parents were 81% female. Adolescents were 62.5% female and had an average age of 15.1 (SD = 2.3). Please see Table 2 for further descriptive information.

**Table 2:** Experiences teaching or counseling about internet safety

***Frequency of teaching or counseling***	**Teachers**	**Clinicians**	**Parents**
	**N(%)**		
*Regularly*	4 (5.2%)	4 (3.6%)	29 (40.3%)
*Sometimes*	16 (20.8%)	61 (55.0%)	42 (58.3%)
*Never*	51 (66.2%)	37 (33.3%)	0 (0%)
*Plan to*	6 (7.8%)	9 (8.1%)	0 (0%)
*Missing data*	0	0	1 (1.4%)

### Age to begin teaching internet safety

**Figure 1:**
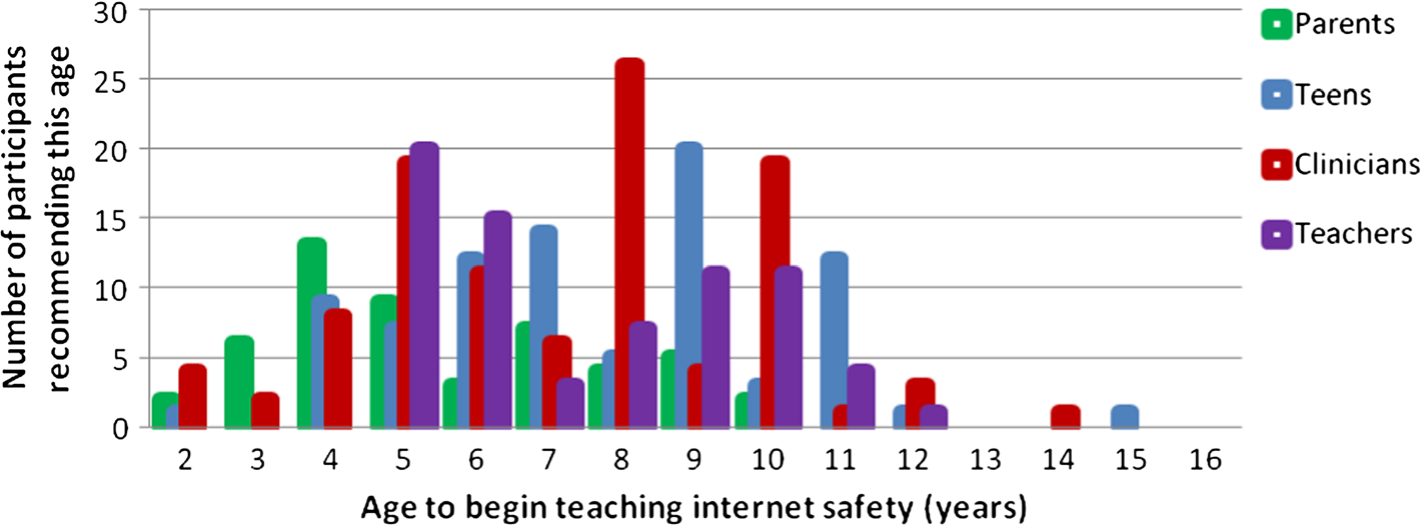
Age to begin teaching internet safety to youth.

The overall mean age at which stakeholders indicated for starting to teach internet safety was 7.2 years (SD = 2.5), range 2-15. Teachers reported that the average age at which internet safety should be taught was 6.9 years (SD = 2.1), while clinicians felt the average age to start teaching this topic should be 7.3 years (SD = 2.4). Parents felt that internet safety education should begin at age 6.6 years (SD = 2.3). There were no statistically significant differences between these groups regarding age to begin teaching internet safety (p = .2). Adolescents reported that internet safety education should begin at age 8.7 years (SD = 2.4). Please see Figure 1 for a summary of recommended ages to begin internet safety education.

### Candidates to teach internet safety

#### Experiences teaching internet safety

Among teachers, 16 (20.8%) reported currently teaching internet safety, 51 (66.2%) had never taught it, and 4 (7.8%) had never taught it but planned to soon. The number of years teaching was not significantly associated with the likelihood to have taught internet safety.

Among clinicians, 3.6% regularly and 55% sometimes counseled patients on internet safety. One-third of clinicians (33.3%) had never counseled or taught patients about internet safety and a few clinicians (8.1%) had no experience with this but planned to begin soon. The number of years in practice was not associated with the likelihood to have taught internet safety (p = .6).

All parents reported discussing online safety with their children either sometimes (58.3%) or regularly (40.3%).

#### Experiences learning internet safety

Adolescents were asked to identify ways in which they had learned about online safety. Adolescents were permitted to select all options that applied. Adolescents selected people including teachers (87.5%), parents (75%), friends (41.7%), siblings (27.1%) and clinicians (11.5%). Some adolescents indicated that they had learned internet safety by being self-taught (27.5%).

#### Willingness to teach internet safety

Teachers uniformly reported supporting online safety education in public schools (100%). Clinicians almost uniformly supported providing online safety education in clinicians’ offices (99.1%).

All groups selected parents as the primary candidate to teach internet safety. Among teachers, 97% ranked parents as their first choice candidate, and 3% ranked teachers as first choice. Among clinicians 97% ranked parents as first choice candidate, and 3% ranked teachers as first choice. Among parents, 96% ranked themselves as first choice candidate, and 4% ranked teachers as first choice. Among adolescents, there was more variety in answers. Most adolescents (74.7%) ranked parents as first choice candidate, 13.8% ranked teachers as first choice, 5.7% ranked law enforcement as first choice, 1.5% ranked community as first choice, 3% ranked churches as first choice and 3% wrote in answers of making a movie related to online safety and making a powerpoint regarding online safety.

## Discussion

The results of this study illustrate several key points regarding promoting safe internet use among youth. Findings suggest general agreement among key stakeholders for teaching internet safety at a young age, and for identifying parents as primary teachers of this topic.

First, our findings regarding the suggested age to begin teaching online safety may seem younger than expected. The suggested age range of 6 to 8 years identified by participants suggests that internet safety education could begin in early grade school, around 1^st^ or 2^nd^ grade. However, given our current society’s focus on technology, it is likely that children are being introduced to computers at ever-younger ages. Data from 2010 suggests that almost 20% of 8 to 10 year olds spend time on social networking sites daily, in the past three years it seems likely that this percentage has grown [20]. Timing safety education with the onset of internet use may allow for the concomitant development of computer skills and safety skills. As with many health teachings such as nutrition or sexual behavior, providing education to children before dangers can arise is a key strategy to help youth integrate these lessons into their lives and prevent negative consequences.

Second, our findings include a general agreement among key stakeholders that parents should hold the primary responsibility for internet safety education. These findings are supported by a recent study in which teachers felt that parents should have the primary role in teaching this topic [27]. Interestingly, we found that while parents *all* reported that they regularly or sometimes teach internet safety, only 75% of adolescents reported hearing from parents on this topic. These conflicting findings may be due to social desirability on the part of parents reporting their teaching efforts, or that teens may underreport their parents counseling efforts as they may not recognize parent attempts to discuss these difficult topics. Previous work has found a similar disconnect between parent and pediatrician reporting of counseling on risk behaviors [28].

Finally, our findings suggest that parents are willing teachers in providing internet safety education, and that many report some experience in this area. However, while parents may be candidates to guide their children’s digital lives, some parents may feel underprepared for the task of instructing their children who have grown up as “digital natives.” Thus, health care providers and public health educators may have an unique opportunity to support parents by providing resources, guidance and support. Pediatricians who see adolescent patients have the opportunity to serve an important and perhaps familiar role. As with many other topics of health supervision including safety, nutrition and fitness, parents are the primary source of education for their children. However, in many of these health topics, clinicians and health educators are trusted sources for parents on how to talk with their children about these issues. Some child health providers may feel untrained or unprepared to answer questions about internet safety or cyberbullying given that these are relatively recent health concerns about which much remains unknown. Pediatricians can use American Academy of Pediatrics guidelines to recommend parental supervision of internet activities, decreasing or eliminating isolated screen time (ie, moving the computer to a public space), and having open discussions about the potential dangers of electronic media [23]. Pediatricians and educators can also partner with schools or other community groups, such as law enforcement, to provide consistent and reinforced messages about internet safety.

Limitations to this study include the regional focus of our data collection. Our study aimed to draw representation of populations of teachers, clinicians, parents and adolescents within our region, the excellent response rates and distribution of participants within each category support that our results are generalizable within our region. However, there are other groups who may engage in teaching internet safety that were not included in this study such as churches and community groups. Second, it is notable that our study did not provide data on what methods would be best to provide internet safety education, this is a logical next step for future study. Third, we did not specify in the context of this study whether online safety should include additional technologies such as cell phones or texting. Fourth, data was collected by self-report, thus recall bias or overestimation of experience or willingness could have impacted our findings. Based on the varied stakeholders included in this study, there was some variation in data collected from each group.

## Conclusions

Technology is now an integral part of life, and thus, part of the health of our patients. Our findings illustrate consensus around several groups with experience and investment in working with children and adolescents that parents should have primary responsibility for teaching internet safety. Our study highlights an opportunity for pediatricians to play a collaborative role with parents, patients and teachers to address the critical topics towards improving internet safety. Given the importance of this topic for today’s youth, it is likely that collaborative efforts are needed to provide consistent education about safety in the digital world.

## Competing interest

The authors declare that they have no competing interest.

## Authors’ contributions

MM conceived of the study, participated in its design and coordination, participated in analysis and wrote the manuscript. KE and KB participated in data collection and helped to draft the manuscript. HY and EC participated in analysis and helped to draft the manuscript. All authors read and approved the final manuscript.

## Authors’ information

MM is an adolescent medicine physician who conducts research on the intersection of technology and health. KE is a medical student interested in pediatrics. KB studied consumer science and is interested in ways to improve internet safety education for youth. HY is a pharmacist and researcher with interest in provision of education to patients and parents. EC is a pediatrician and researcher interested in improving health systems and communication.

## Pre-publication history

The pre-publication history for this paper can be accessed here:

http://www.biomedcentral.com/1471-2458/13/543/prepub

## Supplementary Material

Additional file 1 Survey given to teachers. Click here for file

Additional file 2 Survey given to clinicians. Click here for file

Additional file 3 Survey given to parents. Click here for file

Additional file 4 Survey given to adolescents. Click here for file
