# Supplementary material for: Internet safety education for youth: stakeholder perspectives
Source: BMC Public Health. 2013 Jun 5;13:543. doi: 10.1186/1471-2458-13-543 (PMC3691757; doi:10.1186/1471-2458-13-543)
Supplement: Additional file 1 — Survey given to teachers. [file 1471-2458-13-543-S1.doc]

**Thank you for agreeing to be in our survey. We want this survey to be confidential so please don’t include your name anywhere on the survey.** We’d like to learn a little about you, please answer the following questions:

What type of provider are you? Please check one:

__MD __RN __NP __PA __Other (which?): ___________

What is your field of practice? Please check one:

__Pediatrics __Family practice __OB/GYN __Other(which?): ______

How many years have you been in practice?___________________

**We are interested in your views and experiences regarding online safety education.**

1. What is your experience with counseling patients about online safety? *Please check one*
   ______I have **never** counseled patients on this topic

______I counsel **some** patients on this topic

______I counsel **all** patients on this topic

______I do not counsel patients on this, **but plan to soon**

______Other: *(please explain)*_________________________

1. What is your opinion regarding providing online safety counseling during clinical visits with adolescent patients?

______I am supportive

______I am against it

1. If online safety education were to be provided in health providers’ offices

--At what age should the education begin? __________

--What 3 topics would be most important to counsel teens about regarding online safety?

______________________________________

______________________________________

______________________________________

1. To your knowledge, have your patients encountered any of these situations in which online safety was a concern?

*Please check all that apply*

_______Cyberbullying

_______Unwanted online attention (sexual predation)

_______Identity theft

_______Other: *(please describe)*__________________________

1. In your opinion, whose *primary responsibility* is it to provide online safety education to children and teens? **Please rank in order from 1 to 5**, *with 1 indicating whose primary responsibility it should be*

______Parents

______Teachers

______Law enforcement

______Health care providers

______Community groups

______Churches

______Other: *(please describe)*___________________________

***Thank you for your time and thoughts! Please return the survey to research staff and collect your gift card.***
