# Supplementary material for: Internet safety education for youth: stakeholder perspectives
Source: BMC Public Health. 2013 Jun 5;13:543. doi: 10.1186/1471-2458-13-543 (PMC3691757; doi:10.1186/1471-2458-13-543)
Supplement: Additional file 2 — Survey given to clinicians. [file 1471-2458-13-543-S2.doc]

**Thank you for agreeing to be in our survey. We want this survey to be confidential so please don’t include your name anywhere on the survey.** We’d like to learn a little about you, please answer the following questions:

Are you a: _____Mom _____Dad _____Grandma

_____Grandpa __Other (which?): ___________

How old are your children/grandchildren? ___Infants/Toddlers (0-3 years) ___Early school (4-8 years) ___Preteen (9-12 years)

___Early teen (12-15 years) ____Older teen (16-20 years)

**We are interested in your views and experiences regarding online safety education.**

1. What is your experience with talking to your child/grandchild about online safety? *Please check one*
   __I have **never** talked to my child/grandchild about online safety

__I have talked to my child/grandchild **a few times** about online safety

__I have talked to my child/grandchild **regularly** about online safety

__I have not talked to my child/grandchild, **but plan to soon**

______Other: *(please explain)*_________________________

1. If you have talked with your child/grandchild about online safety, what resources did you use to learn more about this topic? Please list any that you’d like us to know about:

___________________________________________

___________________________________________

1. --At what age should children begin to learn about online safety? __________

--What 3 topics would be most important to counsel children or teens about regarding online safety?

______________________________________

______________________________________

______________________________________

1. To your knowledge, have your children/grandchildren encountered any of these situations in which online safety was a concern?

*Please check all that apply*

_______Cyberbullying

_______Unwanted online attention (sexual predation)

_______Identity theft

_______Other: *(please describe)*__________________________

1. In your opinion, whose *primary responsibility* is it to provide online safety education to children and teens? **Please rank in order from 1 to 5**, *with 1 indicating whose primary responsibility it should be*

______Parents

______Teachers

______Law enforcement

______Health care providers

______Community groups

______Churches

______Other: *(please describe)*___________________________

***Thank you for your time and thoughts! Please return the survey to research staff.***
