# Supplementary material for: Internet safety education for youth: stakeholder perspectives
Source: BMC Public Health. 2013 Jun 5;13:543. doi: 10.1186/1471-2458-13-543 (PMC3691757; doi:10.1186/1471-2458-13-543)
Supplement: Additional file 3 — Survey given to parents. [file 1471-2458-13-543-S3.doc]

**Thank you for agreeing to be in our survey. We want this survey to be confidential so please don’t include your name anywhere on the survey.** We’d like to learn a little about you, please answer the following questions:

Are you a: _____Female _____Male

What grade did you just finish? _______What is your age in years? ______

**We are interested in your views and experiences regarding online safety education.**

1. In what ways have you learned about online safety?

*Please check as many as apply*

___From my friends

___From my siblings

___From my parents

___Teachers at school

___Health care professional (doctor or nurse or therapist)

___I have taught myself about online safety

___Other: (please explain) ____________________________

Are there resources you have used to learn about this topic? __Yes __No

Please list any websites or articles/brochures that you’d like us to know about:__________________________________________

____________________________________________________

1. At what age should children begin to learn about online safety? __________
2. What 3 topics would be most important to counsel children or teens about regarding online safety?

________________________________________________

________________________________________________

________________________________________________

1. In your opinion, whose *primary responsibility* is it to provide online safety education to children and teens? **Please rank in order from 1 to 5**, *with 1 indicating whose primary responsibility it should be*

______Parents

______Teachers

______Law enforcement

______Health care providers

______Community groups

______Churches

______Other: *(please describe)*___________________________

1. In your opinion, **how** should online safety be taught?

______With an Internet site

______By brochure or book

______Someone should teach it to me

______Other: _____________________________________

1. Have you ever encountered any of these situations in which online safety was a concern? *Please check all that apply*

_______Cyberbullying

_______Unwanted online attention (sexual predation)

_______Identity theft

_______Other: *(please describe)*__________________________

***Thank you for your time and thoughts! Please return the survey to research staff.***
