# Supplementary material for: Internet safety education for youth: stakeholder perspectives
Source: BMC Public Health. 2013 Jun 5;13:543. doi: 10.1186/1471-2458-13-543 (PMC3691757; doi:10.1186/1471-2458-13-543)
Supplement: Additional file 4 — Survey given to adolescents. [file 1471-2458-13-543-S4.doc]

**Thank you for agreeing to be in our survey. We want this survey to be confidential so please don’t include your name anywhere on the survey.** We’d like to learn a little about you, please answer the following questions:

Check your primary teaching site: __Elementary___Middle___High

If you teach a specific subject(s) it is primarily in the area of:

___Social studies, English, languages

___Science, math, psychology

___Computers/technology

___Art, music, physical education

___Other:______________

___I don’t teach a particular subject area

How many years have you been teaching?_____________________

**We are interested in your views regarding online safety education.**

1. What is your experience teaching online safety education? *Please check one*
   ______I have never taught this topic

______I currently teach this topic

______I don’t teach this topic but plan to soon

______Other: *(please explain)*_________________________

1. What is your opinion regarding providing online safety education in public schools?

______I am supportive

______I am against it

1. If online safety education were to be provided in all public schools

--At what grade level should the education begin? __________

--In what class subjects should online safety be taught?___________________________________________

--What 3 topics would be most important to include in a class regarding online safety?

______________________________________

______________________________________

______________________________________

1. Has your school encountered any of these situations in which online safety of students was a concern? *Please check all that apply*

_______Cyberbullying

_______Unwanted online attention (sexual predation)

_______Identity theft

_______Other: *(please describe)*__________________________

1. In your opinion, whose *primary responsibility* is it to provide online safety education to children and teens? Please rank in order from 1 to 5, *with 1 indicating whose primary responsibility it should be*

______Parents

______Teachers

______Law enforcement

______Health care providers

______Community groups

______Churches

______Other: *(please describe)*___________________________

***Thank you for your time and thoughts! Please return the survey to research staff and collect your gift card.***
